# Well-Defined Supported ZnO_*x*_ Species: Synthesis, Structure, and Catalytic Performance in Nonoxidative Dehydrogenation of C_3_–C_4_ Alkanes

**DOI:** 10.1021/acs.accounts.4c00011

**Published:** 2024-04-09

**Authors:** Shanlei Han, Dan Zhao, Evgenii V. Kondratenko

**Affiliations:** † Leibniz-Institut für Katalyse e.V., Albert-Einstein-Str. 29a, 18059 Rostock, Germany

## Abstract

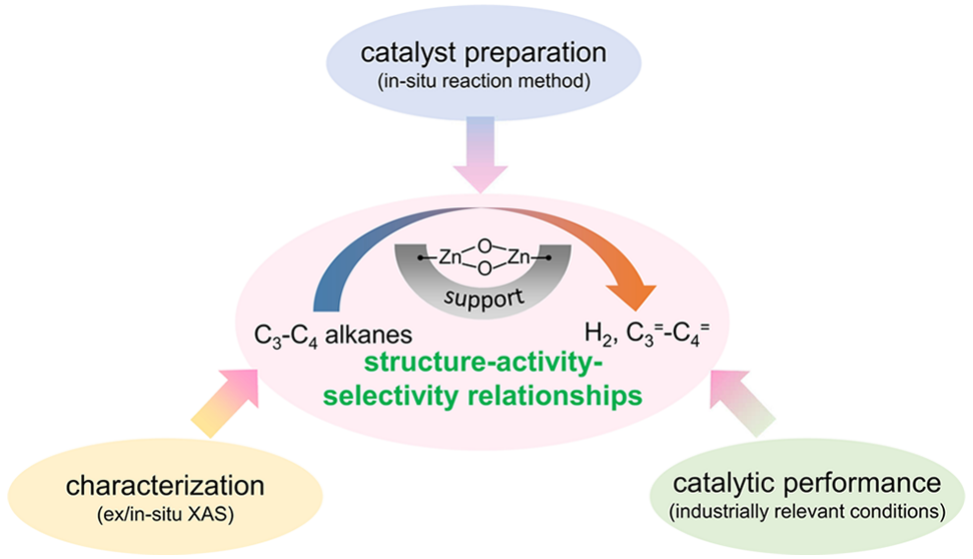

Zinc oxide (ZnO) is a multipurpose material and finds its applications in various fields such as rubber manufacturing, medicine, food additives, electronics, etc. It has also been intensively studied in photocatalysis due to its wide band gap and environmental compatibility. Recently, heterogeneous catalysts with supported ZnO_*x*_ species have attracted more and more attention for the dehydrogenation of propane (PDH) and isobutane (iBDH) present in shale/natural gas. The olefins formed in these reactions are key building blocks of the chemical industry. These reactions are also of academic importance for understanding the fundamentals of the selective activation of C–H bonds. Differently structured ZnO_*x*_ species supported on zeolites, SiO_2_, and Al_2_O_3_ have been reported to be active for nonoxidative dehydrogenation reactions. However, the structure–activity–selectivity relationships for these catalysts remain elusive. The main difficulty stems from the preparation of catalysts containing only one kind of well-defined ZnO_*x*_ species.

In this Account, we describe the studies on PDH and iBDH over differently structured ZnO_*x*_ species and highlight our approaches to develop catalysts with controllable ZnO_*x*_ speciation relevant to their performance. Several methods, including (i) the in situ reaction of gas-phase metallic Zn atoms with OH groups on the surface of supports, (ii) one-pot hydrothermal synthesis, and (iii) impregnation/anchoring methods, have been developed/used for the tailored preparation of supported ZnO_*x*_ species. The first method allows precise control of the molecular structure of ZnO_*x*_ through the nature of the defective OH groups on the supports. Using this method, a series of ZnO_*x*_ species ranging from isolated, binuclear to nanosized ZnO_*x*_ have been successfully generated on different SiO_2_-based or ZrO_2_-based supports as demonstrated by complementary ex/in situ characterization techniques. Based on kinetic studies and detailed characterization results, the intrinsic activity (Zn-related turnover frequency) of ZnO_*x*_ was found to depend on its speciation. It increases with an increasing number of Zn atoms in a Zn_*m*_O_*n*_ cluster from 1 to a few atoms (less than 10) and then decreases strongly for ZnO_*x*_ nanoparticles. The latter promote the formation of undesired C_1_–C_2_ hydrocarbons and coke, resulting in lower propene selectivity in comparison with the catalysts containing only ZnO_*x*_ species ranging from isolated to subnanometer Zn_*m*_O_*n*_ clusters. In addition, the strategy for improving the thermal stability of ZnO_*x*_ species and the consequences of mass-transport limitations for DH reactions were also elucidated. The results obtained allowed us to establish the fundamentals for the targeted preparation of well-structured ZnO_*x*_ species and the relationships between their structures and the DH performance. This knowledge may inspire further studies in the field of C–H bond activation and other reactions, in which ZnO_x_ species act as catalytically active sites or promoters, such as the dehydroaromatization of light alkanes and the hydrogenation of CO_2_ to methanol.

## Key Reference

- ZhaoD.; GaoM.; TianX.; DoronkinD. E.; HanS.; GrunwaldtJ.-D.; RodemerckU.; LinkeD.; YeM.; JiangG.; JiaoH.; KondratenkoE. V.Effect of Diffusion Constraints and ZnO_x_ Speciation on Nonoxidative Dehydrogenation of Propane and Isobutane over ZnO-Containing Catalysts. ACS Catal.2023, 13, 3356–336910.1021/acscatal.2c05704.^1^*The selectivity of isobutene over ZnO*_*x*_*/deAl-Beta catalysts is higher than that over the ZnO*_*x*_*/S-1 catalyst due to the absence of diffusion constraints. Subnanometer three-dimensional ZnO*_*x*_*clusters in deAl-Beta reveal higher activity than binuclear ZnO*_*x*_*species in S-1*.
- ZhaoD.; GuoK.; HanS.; DoronkinD. E.; LundH.; LiJ.; GrunwaldtJ.-D.; ZhaoZ.; XuC.; JiangG.; KondratenkoE. V.Controlling Reaction-Induced Loss of Active Sites in ZnO_x_/Silicalite-1 for Durable Nonoxidative Propane Dehydrogenation. ACS Catal.2022, 12, 4608–461710.1021/acscatal.1c05778.^2^*Promoting silicalite-1 with MgO favors the formation of Zn–O–Mg structures with an improved resistance against reduction under propane dehydrogenation conditions. Consequently, the loss of zinc is hindered*.
- ZhaoD.; TianX.; DoronkinD. E.; HanS.; KondratenkoV. A.; GrunwaldtJ.-D.; PerechodjukA.; VuongT. H.; RabeahJ.; EckeltR.; RodemerckU.; LinkeD.; JiangG.; JiaoH.; KondratenkoE. V.In situ Formation of ZnO_x_ Species for Efficient Propane Dehydrogenation. Nature2021, 599, 234–23810.1038/s41586-021-03923-334759363 PMC8580824.^3^*Active ZnO*_*x*_*sites are formed in situ through a reaction between gas-phase Zn atoms and defective OH groups of supports. The best-performing catalyst shows about 3 times higher propene productivity compared with that of a commercial analogue of K-CrO*_*x*_*/Al*_*2*_*O*_*3*_*with close propene selectivity*.
- HanS.; ZhaoD.; OtroshchenkoT.; LundH.; BentrupU.; KondratenkoV. A.; RockstrohN.; BartlingS.; DoronkinD. E.; GrunwaldtJ.-D.; RodemerckU.; LinkeD.; GaoM.; JiangG.; KondratenkoE. V.Elucidating the Nature of Active Sites and Fundamentals for their Creation in Zn-Containing ZrO_2_-Based Catalysts for Nonoxidative Propane Dehydrogenation. ACS Catal.2020, 10, 8933–894910.1021/acscatal.0c01580.^4^*Isolated tricoordinated Zn*^*2+*^*O*_*x*_*species on the surface of TiZrO*_*x*_*show high selectivity to propene in propane dehydrogenation. Their intrinsic activity is governed by a synergy effect between ZnO, ZrO*_*2*_*,and TiO*_*2*_*that is relevant to accelerating hydrogen formation*.

## 1 Introduction

Propene and butenes are important building blocks in the chemical industry due to their wide-range applications.^5−7^ They are currently produced from various oil- and coal-based feedstocks. Propane and isobutane, which are present in natural/shale gas, are alternative economic raw materials. In this context, the nonoxidative dehydrogenation of propane (PDH) and isobutane (iBDH) to the corresponding olefins has become the basis of several large-scale processes using CrO_*x*_- or Pt-containing catalysts.^5−8^ However, the catalysts used suffer from the environmental incompatibility of Cr(VI) compounds and the high cost of Pt. Another shortcoming of Pt-based catalysts is the need to use ecologically harmful chlorine or chlorine-containing hydrocarbons to redisperse Pt particles during catalyst regeneration.

Against this background, various materials have been developed.^5−8^ The most promising candidates are catalysts with supported GaO_*x*_,^9−12^ VO_*x*_,^13−17^ or CoO_*x*_^18−24^ species. Considering the cost and environmental friendliness of zinc, ZnO_*x*_-based catalysts are of particular interest. Both bulk^25,26^ and supported catalysts with different ZnO_*x*_ species on the surface of zeolites,^27^ SiO_2_^28^ and Al_2_O_3_,^29^ have been tested but showed industrially unattractive performance. Our group^1,3^ developed an economical and efficient method for the preparation of Zn-containing catalysts using commercially available ZnO and different oxidic supports. This method allows the generation of supported ZnO_*x*_ with a certain structure ranging from isolated species to ZnO nanoparticles and thus structure–activity–selectivity relationships can be established. The optimized catalyst showed appealing propene productivity and selectivity under industrially relevant conditions. In this Account, we compare our achievements to current relevant developments. Special emphasis is placed on the kind/structure of the supported ZnO_*x*_ species, the mechanism of their formation to enable targeted catalyst preparation, and the structure–activity–selectivity relationships. The application potential of the developed catalysts is demonstrated by comparing their performance in PDH with that of other state-of-the-art catalysts. Finally, the remaining challenges and opportunities of ZnO_*x*_-based catalysts in alkane dehydrogenation reactions are discussed.

## 2 Setting the Scene of ZnO_*x*_-Containing Catalysts in C_3_H_8_ Dehydrogantion

### 2.1 Benchmarking State-of-the-Art PDH Catalysts

**Figure 1:**
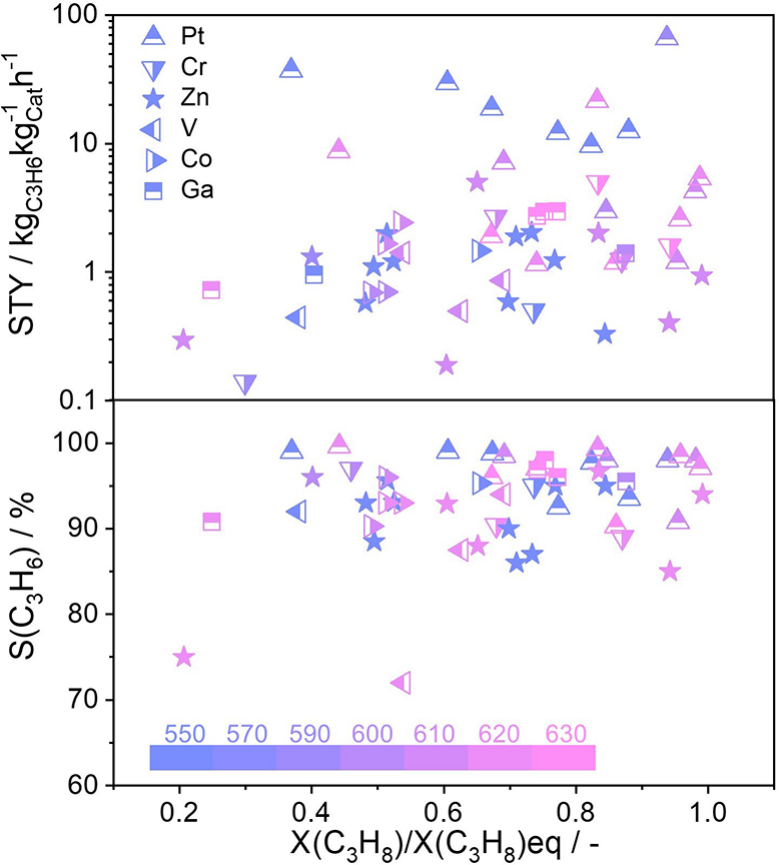
Comparison of the space–time yield of propene formation (STY(C_3_H_6_)) and propene selectivity (S(C_3_H_6_)) determined over various catalysts in the PDH reaction under different reaction conditions. The values and the corresponding references are available in Table S1 in the Supporting Information.

The relevance of studies on the development of Zn-containing catalysts for alkane dehydrogenation is illustrated by Figure 1, which shows the space–time yield of propene formation (STY(C_3_H_6_)) and propene selectivity (S(C_3_H_6_)) over different catalysts. For a fair comparison, it is highly important to consider the equilibrium propane conversion (X(C_3_H_8_)eq), which is affected by the feed composition and reaction temperature. For this purpose, we calculated the ratio of the reported conversion (X(C_3_H_8_)) to X(C_3_H_8_)eq, i.e., X(C_3_H_8_)/X(C_3_H_8_)eq). Industrially attractive catalysts should have high STY(C_3_H_6_) and (S(C_3_H_6_)) at X(C_3_H_8_)/X(C_3_H_8_)eq as close to 1 as possible.

Given their use in large-scale PDH and iBDH processes, it is not surprising that Pt-based materials show a high STY(C_3_H_6_) (Figure 1). ZnO_*x*_-based catalysts are slightly less active but outperform most Pt-free catalysts. They are also attractive in terms of selectivity. Propene selectivity values of over 90% have been obtained at (X(C_3_H_8_)/X(C_3_H_8_)eq) close to 1 (Figure 1).

### 2.2 Industrial Relevance of ZnO_*x*_-Based Catalysts

**Figure 2:**
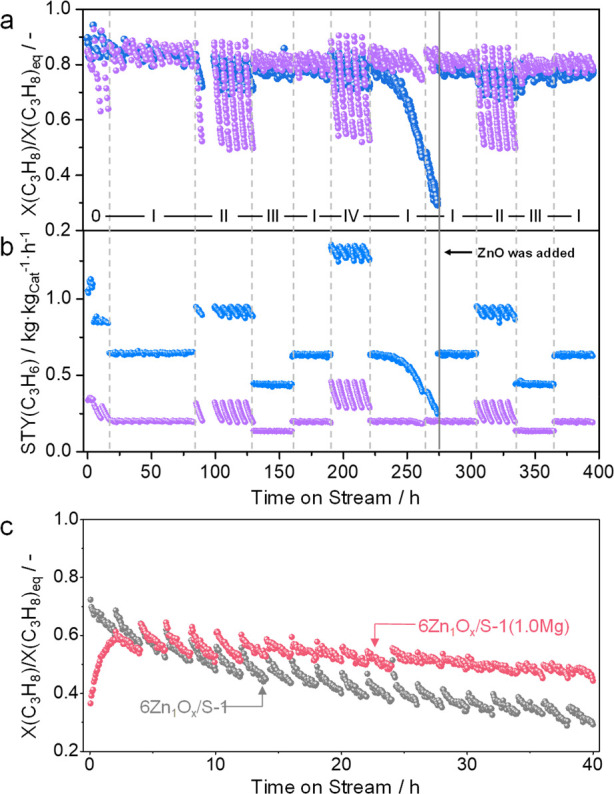
Time-on-stream profiles of (a) X(C_3_H_8_)/X(C_3_H_8_)_eq_ and (b) STY(C_3_H_6_) determined over ZnO//ZnO-S-1_3 (solid blue balls, 0.09 g of ZnO and 0.09 g of ZnO-S-1_3) and commercial-like K-CrO_*x*_/Al_2_O_3_ (solid purple balls). Reprinted with permission from ref (3). Copyright 2021 Springer Nature. (c) Time-on-stream profiles of X(C_3_H_8_)/X(C_3_H_8_)_eq_ determined over 6Zn_1_O_*x*_/S-1 and 6Zn_1_O_*x*_/S-1(1.0Mg) at 550 °C. Reprinted with permission from ref (2). Copyright 2022 American Chemical Society. Reaction conditions for (a, b) (0): C_3_H_8_:H_2_:N_2_ = 2:1:2, 10, or 6 mL·min^–1^; 6 mL min^–1^ for (I): C_3_H_8_:H_2_:N_2_ = 2:1:2; (II) C_3_H_8_:N_2_ = 2:3; (III) C_3_H_8_:H_2_:N_2_ = 2:2:1; (IV) C_3_H_8_:N_2_ = 7:3 and for (c) C_3_H_8_:N_2_ = 2:3, 10 min·min^–1^.

Catalyst durability, i.e., the ability of a catalyst to recover its performance after regeneration, is a key property relevant to industrial applications. The results from ref (3) are particularly noteworthy because of the exceptional catalyst operation time of about 400 h under different industrially relevant conditions. The test using ZnO/S-1_3 with an upstream ZnO layer (ZnO//ZnO/S-1_3) and a commercial-like K-CrO_*x*_/Al_2_O_3_ catalyst consisted of 64 PDH/regeneration cycles. These catalysts were tested in parallel in the same setup. The space velocity was adjusted for each catalyst to achieve an X(C_3_H_8_)/X(C_3_H_8_)eq value of between 0.8 and 0.9 (Figure 2a). The ZnO//ZnO/S-1_3 catalyst showed about 3 times higher STY(C_3_H_6_) than K-CrO_*x*_/Al_2_O_3_ (Figure 2b) with a similar average propene selectivity, i.e., 91.9 versus 93.1%. Both catalysts were deactivated during the PDH cycle due to coke formation but recovered their activity after oxidative regeneration. However, the activity of ZnO//ZnO/S-1_3 decreased dramatically after the first 250 h on the propane stream. The decrease was explained by the loss of ZnO_*x*_ species in the form of metallic Zn formed by the reduction of ZnO_*x*_ in C_3_H_8_/C_3_H_6_/H_2_ based on the following experimental observations: (i) the top layer of ZnO disappeared after 273 h on the propane stream, (ii) ZnO_*x*_ species catalyze PDH, and (iii) the catalyst recovered its initial activity after the addition of a fresh ZnO layer and performed durably for the next 150 h. The mechanism of the ZnO_*x*_ species formation is explained in section 3. Coke formation was also concluded to be responsible for catalyst deactivation during a PDH cycle with increasing time on the propane stream. This undesired product can be easily removed by an oxidative catalyst treatment without changing the activity of the supported ZnO_*x*_ species. The loss of Zn is an irreversible process. In our separate study, we have demonstrated that this unwanted process could be partially hindered through increasing the thermal stability of ZnO_*x*_ species by modifying the silicalite-1 support with MgO (Figure 2c).^2^ Zn–O–Mg bonds in the modified catalyst (6Zn_1_O_*x*_/S-1(1.0Mg)) have lower reducibility than Zn–O–Si bonds. Compared with the unmodified 6Zn_1_O_*x*_/S-1 catalyst, which lost about 48% of its initial activity during 20 PDH/regeneration cycles, the modified catalyst lost about 28%. This is due to the different ability of these catalysts to lose Zn under reaction conditions. The loading of Zn in 6Zn_1_O_*x*_/S-1 and 6Zn_1_O_*x*_/S-1(1.0Mg) decreased by 29 and 15%, respectively. Further studies in this direction are urgently needed to make Zn-based catalysts ready for commercial applications.

## 3 Supported ZnO_*x*_ Structures and Their Preparation Methods

**Figure 3:**
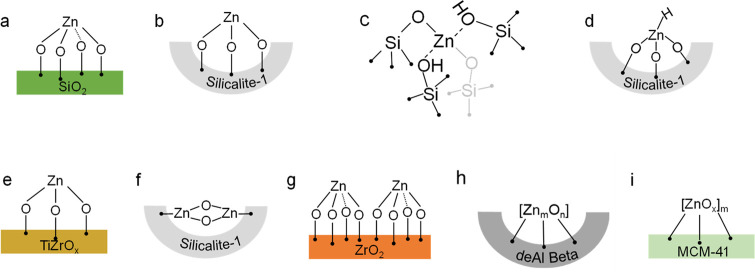
Suggested structures of ZnO_*x*_ species on the surfaces of various supports.

Since the structure of supported ZnO_*x*_ species determines catalyst activity and product selectivity, it is very important to provide the fundamentals relevant for the targeted preparation of catalysts with uniformly structured species. This knowledge is relevant not only to PDH and iBDH but also to alkane dehydroaromatization and CO_2_ hydrogenation to methanol, where ZnO_*x*_ species are active sites and/or promoters. The reported ZnO_*x*_ structures are summarized in Figure 3. They depend on the kind of support and catalyst preparation methods. For example, Schweitzer et al.^28^ used a strong electrostatic adsorption method to prepare four-coordinate isolated Zn_1_O_*x*_ supported on amorphous SiO_2_ (Figure 3a). Their structure was determined by X-ray absorption spectroscopy (XAS). According to this method, OH groups on the support surface become positively or negatively charged when the pH of the solution was lower or higher than the point of zero charge (PZC). Thus, anions or cations of metal precursors can be readily adsorbed onto the support surface.^30^ Siliceous zeolites, which have a special topology, have also been applied as supports for ZnO_*x*_ species. The types of Zn precursors and their complexing agents are the key factors for the formation of well-structured ZnO_*x*_ species. For example, we prepared isolated Zn_1_O_*x*_ sites on the surface of silicalite-1 (S-1) by a one-pot hydrothermal method using ethylenediamine (EDA) as a ligand to stabilize Zn^2+^ in the parent gel (Figure 3b).^2^ Their structure was determined by XAS and X-ray diffraction. The presence of EDA is important to hinder the formation of MO_4_ tetrahedra, which can replace SiO_4_ or AlO_4_ in the zeolite framework, and thus MO_x_ species could be formed on the surface of the zeolite.^31^ Su et al.^32^ prepared Zn-MFI materials with Zn cations implanted into the framework of the zeolite by a one-pot hydrothermal method using Zn(C_6_H_12_O_7_)_2_ as the Zn precursor. DFT calculations predict the formation of isolated Zn_1_O_*x*_ species with two Zn–O–Si and two Zn···OH–Si bonds (Figure 3c). Song and co-workers^14^ found that small Zn_*n*_O_*x*_ clusters were formed when Zn(acac)_2_ was used as the Zn precursor, while isolated Zn–OH species were formed when Cu(acac)_2_ was co-added during catalyst preparation. The isolated species are precursors of [ZnH]^+^ sites formed under PDH conditions (Figure 3d).^33^

ZrO_2_-based materials have also been used as supports for ZnO_*x*_ species deposited by a wet impregnation method. Their structure depends on the kind of metal oxide dopant in the lattice of ZrO_2_.^4^ Isolated Zn_1_O_*x*_ species identified by XAS were formed on the surface of TiZrO_*x*_ when the Zn loading was below 4 wt % (Figure 3e). At the same Zn loading, isolated Zn_1_O_*x*_ and ZnO nanoparticles were found to coexist on the surface of pure ZrO_2_ or ZrO_2_ doped with Y^3+^, La^3+^, or Ce^4+^. The necessity of the coexistence of ZrO_2_ and TiO_2_ in the support for the preparation of highly dispersed ZnO_*x*_ was demonstrated in a separate study.^34^ Small ZnO_*x*_ clusters with 1 to 3 Zn atoms were produced when rutile TiO_2_ was impregnated with an aqueous solution of Zn(NO_3_)_2_·6H_2_O and ZrO(NO_3_)_2_·*x*H_2_O.^34^

**Figure 4:**
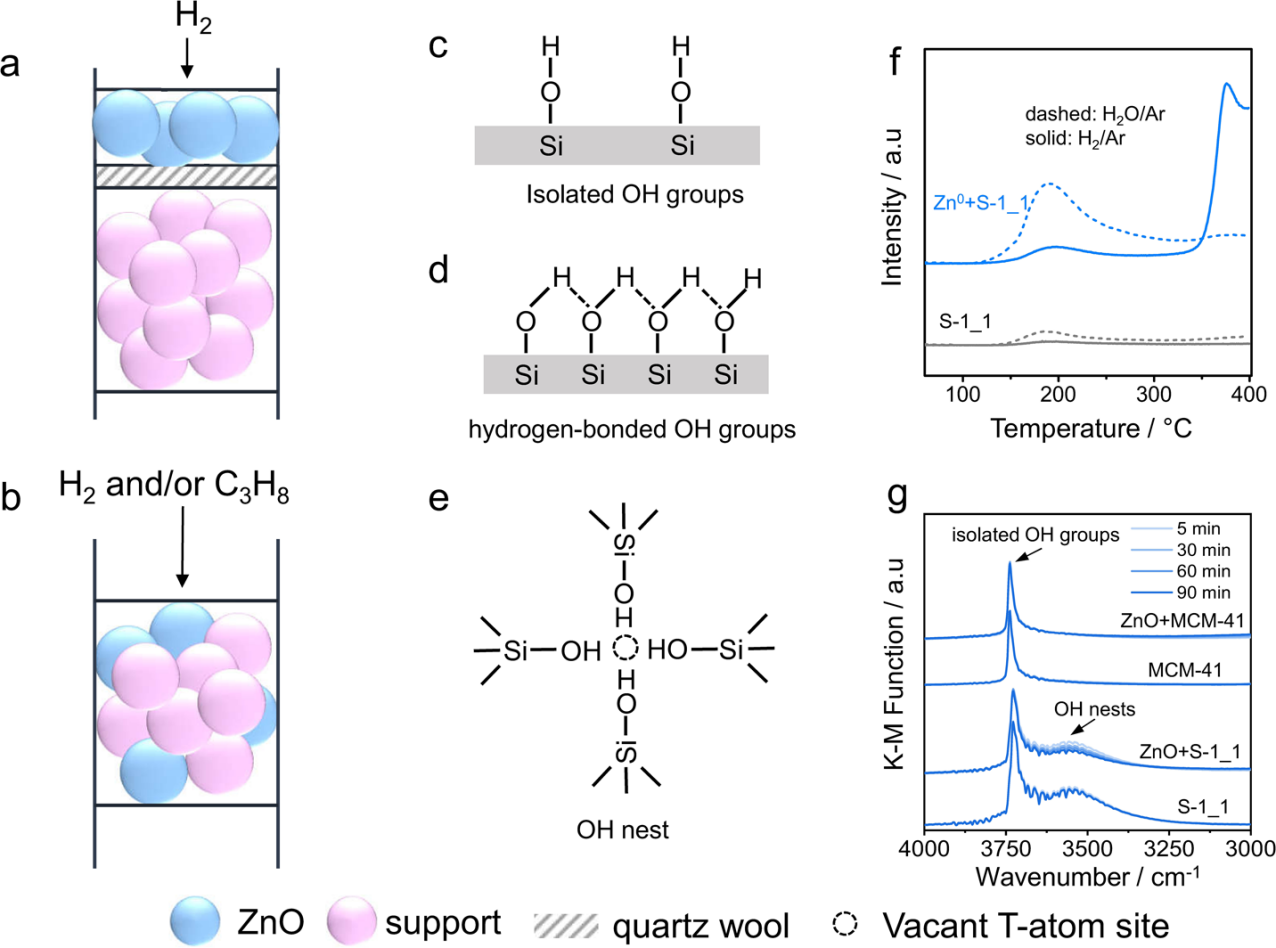
Preparation of supported ZnO_*x*_ species upon reductive treatment of (a) separated ZnO and support layers or (b) a physical ZnO-support mixture. Structures of (c) isolated OH groups, (d) hydrogen-bonded OH groups, and (e) OH nests in zeolites. (f) Profiles of H_2_ and H_2_O formed during temperature-programmed heating of S-1 or a physical mixture of metallic Zn and S-1 in Ar. (g) In situ DRIFT spectra recorded during the treatment of bare supports or their mixtures with ZnO in a flow of 50 vol %H_2_/Ar at 550 °C. (f, g) reprinted with permission from ref (3). Copyright 2021 Springer Nature.

A simple method using only solid materials, i.e., ZnO and zeolites or metal oxides, has been developed by our group.^3^ This strategy is environmentally friendly because it produces neither solid nor liquid waste. The catalyst components are loaded into a tubular furnace either in the form of two separate layers, with ZnO as the upstream layer, or as a physical mixture (Figure 4a,b). They are treated above 500 °C in a flow containing a reducing agent, e.g., H_2_ or C_3_H_8_. Supported ZnO_*x*_ species are formed according to the following three steps: (i) ZnO is reduced to Zn^0^; (ii) the formed Zn^0^ atoms escape to the gas phase, and (iii) the Zn^0^ atoms are then oxidized by support OH groups to form supported ZnO_*x*_.^3^ Control tests with a physical mixture of S-1 and metallic Zn showed the formation of H_2_ (Figure 4f) by the consumption of OH groups by Zn^0^ (Figure 4g).

The kind of OH groups and the support morphology appear to be the most decisive factors affecting the structure of the ZnO_*x*_ species. OH groups can be isolated (Figure 4c) or hydrogen-bonded (Figure 4d). The latter with a different structure in zeolites is called OH nests (Figure 4e). The isolated OH species are stable up to 800 °C.^35^ The other two kinds of OH groups can be transformed into isolated OH groups at high temperatures.^36^ Using S-1 with abundant OH nests as a support, Zn_2_O_*x*_ species (Figure 3f) with two bridged oxygen species were prepared.^1,3^ Small Zn_*m*_O_*n*_ clusters (Figure 3h) were formed on the surface of the deAl-Beta zeolite, which also has OH nests but larger pores. In comparison with S-1, Zn_2_O_*x*_ with a different geometry was generated on the surface of ZrO_2_ (Figure 3g).^1^ Supports possessing only isolated OH groups, such as MCM-41, favor the formation of ZnO nanoparticles (NPs) (Figure 3i).

## 4 Identification of Differently Structured ZnO_*x*_ Species by XAS

X-ray absorption spectroscopy (XAS) is one of the most powerful techniques for providing information on the local structure and electronic properties of supported metal/metal oxide species in the short range (up to 5 Å).^37^ The oxidation state of metals and the average coordination number (CN) of metal–metal and/or metal–oxygen can be obtained from X-ray absorption near-edge structure (XANES) and extended X-ray absorption fine structure (EXAFS) spectra, respectively.

**Figure 5:**
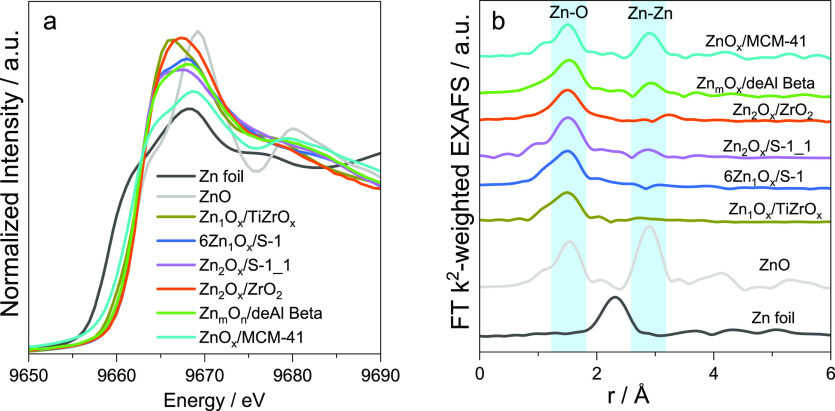
(a) XANES and (b) FT EXAFS spectra of different ZnO_*x*_ species.

The oxidation state of zinc in supported ZnO_*x*_ species depends on their structure or the kind of support. Zn^2+^ is present in Zn_1_O_*x*_ and Zn_2_O_*x*_ sites as well as in Zn_*m*_O_*n*_ clusters formed with the participation of zeolite OH nests, as inferred from the similarity of their XANES spectra with the spectrum of ZnO (Figure 5a). The oxidation state of zinc in ZnO_*x*_ NP on the surface of MCM-41 should be between 0 and +2.

The local structure of the supported ZnO_*x*_ species (Figure 3e–i) was determined through fitting EXAFS spectra.^1−4^ Zn_1_O_*x*_ species should be present in 6Zn_1_O_*x*_/S-1,^2^ 6Zn_1_O_*x*_/S-1(1.0Mg),^2^ and Zn_1_O_*x*_/TiZrO_*x*_^4^ because only the backscattering of oxygen atoms (uncorrected distance around 1.5 Å) was fitted with an average CN of Zn–O of 3 (Figure 5b and Table 1). The Zn–Zn scattering at about 2.9 Å is visible in the EXAFS spectra of Zn_2_O_*x*_/S-1_1 and Zn_2_O_*x*_/ZrO_2_. The fitted CNs of Zn–O and Zn–Zn in Zn_2_O_*x*_/S-1_1 are 3 and 1, respectively.^1,3^ Thus, this catalyst should possess binuclear Zn_2_O_*x*_ species. However, the CNs of Zn–O and Zn–Zn determined from the EXAFS spectrum of Zn_2_O_*x*_/ZrO_2_ are 4 (in total) and 1, respectively. Moreover, the Zn–Zn distance is about 0.2 Å longer than that in Zn_2_O_*x*_/S-1_1. Thus, binuclear Zn_2_O_*x*_ species should be stabilized on the surface of ZrO_2_ but with a different geometry in comparison with that of Zn_2_O_*x*_ on the surface of S-1.^1^ As the intensity of the Zn–Zn shell in the EXAFS spectrum of ZnO_*x*_/deAl-Beta is more pronounced than in the EXAFS spectrum of Zn_2_O_*x*_/S-1_1, the average Zn–Zn CN in the former Zn-containing species should be higher. The fitting results suggest the presence of small 3-dimensional Zn_*m*_O_*n*_ clusters.^1^ The average CN of Zn–Zn in ZnO_*x*_ species in ZnO_*x*_/MCM-41 is 8.4, suggesting the presence of NPs.^3^

**Table 1:** EXAFS Fitting Results

catalyst	shell	CN	distance/(Å)	σ^2^/(10^–3^ Å^2^)	δE_0_/(eV)	ρ/(%)
Zn_1_O_*x*_/TiZrO_*x*_	Zn–O	2.6 ± 0.2	1.99 ± 0.01	8.0 ± 1.7	3.0 ± 0.7	0.4
	Zn–Zn	n.f.^a^	n.f.^a^	n.f.^a^		
6Zn_1_O_*x*_/S-1	Zn–O	3.4 ± 0.2	1.97 ± 0.01	8.3 ± 1.2	2.3 ± 0.7	0.3
	Zn–Zn	n.f.^a^	n.f.^a^	n.f.^a^		
6Zn_1_O_*x*_/S-1(1.0Mg)	Zn–O	3.3 ± 0.3	1.98 ± 0.01	9.2 ± 1.5	1.9 ± 0.9	0.5
	Zn–Zn	n.f.^a^	n.f.^a^	n.f.^a^		
Zn_2_O_*x*_/S-1_1	Zn–O	2.9 ± 0.2	1.97 ± 0.01	7.3 ± 1.6	4.7 ± 0.6	0.2
	Zn–Zn	1.3 ± 0.5	3.32 ± 0.07	7.3 ± 1.6		
Zn_2_O_*x*_/ZrO_2_	Zn–O	2.9 ± 0.1	2.00 ± 0.04	7.3^b^	–0.84 ± 1.2	2.0
	Zn–Zn	1.1 ± 0.5	3.52 ± 0.03	7.3^b^		
	Zn–O	1.2 ± 0.4	3.24 ± 0.02	7.3^b^		
Zn_*m*_O_*n*_/deAl-Beta	Zn–O	2.9 ± 0.1	1.98 ± 0.01	7.3^b^	3.9 ± 0.6	0.3
	Zn–Zn	6.1 ± 2.1	3.30 ± 0.02	18.7 ± 4.1		
ZnO_*x*_/MCM-41	Zn–O	2.5 ± 0.2	1.95 ± 0.01	7.3^b^	2.6 ± 1.1	0.9
	Zn–Zn	8.4 ± 2.4	3.25 ± 0.01	14.8 ± 3.4		

a n.f. means not fitted.

b The value was fixed to 7.3, which was often used for a measurement at room temperature.

**Figure 6:**
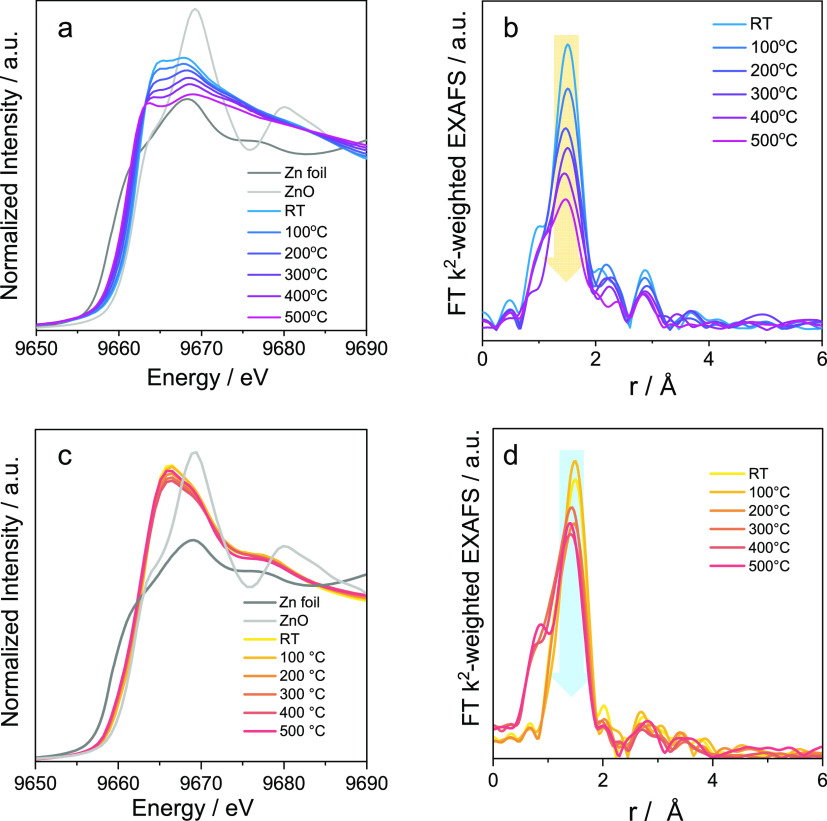
(a, c) XANES and (b, d) EXAFS spectra of (a, b) Zn_2_O_*x*_/S-1_1 or (c, d) Zn_1_O_*x*_/TiZrO_*x*_ treated in 5 vol % H_2_/He or in 20 vol % H_2_/He in the temperature range of 100 to 500 °C. (a, b) and (c, d) Reprinted with permission from refs (3) and (4), respectively. Copyright 2021 Springer Nature and copyright 2020 American Chemical Society, respectively.

Importantly, the local structure of Zn_2_O_*x*_ species on the surface of S-1 and the oxidation state of zinc change under reducing conditions as revealed by in situ XAS measurements using an H_2_-containing feed.^3^ As the temperature increased from 100 to 500 °C, the absorption edge in the XANES spectra shifted to lower values characteristic of metallic Zn^0^ due to Zn_2_O_*x*_ reduction (Figure 6a). Consistently, the intensity of the Zn–Zn shell at 2.9 Å (uncorrected distance) in the EXAFS spectra decreased when the sample was heated to 200 °C and did not change further when the temperature was increased further to 500 °C (Figure 6b). Based on the fitting results at 500 °C, it was concluded that the CN of Zn–O decreased from 3 to 2 while the CN of Zn–Zn did not change. Thus, partially reduced Zn_2_O_*x*_ species are true active sites involved in propane dehydrogenation.^3^

In contrast to Zn_2_O_*x*_/S-1_1, no obvious change in the oxidation state of Zn^2+^ in Zn_1_O_*x*_ species on the surface of TiZrO_*x*_ could be seen from in situ XANES spectra (Figure 6c) recorded during catalyst heating to 500 °C in H_2_.^4^ The Zn_1_O_*x*_ species should be stable against sintering, as no Zn–Zn scattering could be observed in the EXAFS spectra at all temperatures (Figure 6d).

## 5 Factors Affecting Catalyst Performance

### 5.1 Rate-Determining Step of the PDH Reaction over Different Catalysts

The overall rate of a chemical reaction is typically determined by the slowest step, called the rate-determining step (RDS). The widely accepted mechanistic concepts of PDH consider five elementary steps: (i) adsorption of propane, (ii) cleavage of a C–H bond in propane to yield a surface C_3_H_7_ intermediate, (iii) cleavage of a C–H bond in C_3_H_7_ to yield adsorbed propene, (iv) desorption of propene, and (v) formation of H_2_ through the recombination of surface H species.^38^ Based on kinetic analysis and DFT calculations, Chen et al. concluded that C–H bond activation, either step ii or iii, is the RDS in PDH over catalysts with supported Pt nanoparticles regardless of their sizes.^38^ Lercher et al. reported that the RDS in PDH over Ga-BEA is C–H bond activation at a propane partial pressure of about 2 mbar. The formation of H_2_ becomes the RDS when the propane partial pressure is higher than 100 mbar.^39^ This process also limits the PDH reaction over bulk ZrO_2_-based catalysts as concluded from DFT calculations.^40,41^ Supported Rh NPs on the surface of such catalysts accelerate H_2_ formation.^42^ DFT calculations predict that the RDS in PDH over 6Zn_1_O_*x*_/S-1 is the formation of H_2_, while the first C–H bond cleavage in C_3_H_8_ is the slowest step over Zn_2_O_*x*_/S-1_1.^3^ The latter reaction is also the RDS in PDH over isolated Zn_1_O_*x*_ species on the surface of amorphous SiO_2_.^28^

**Figure 7:**
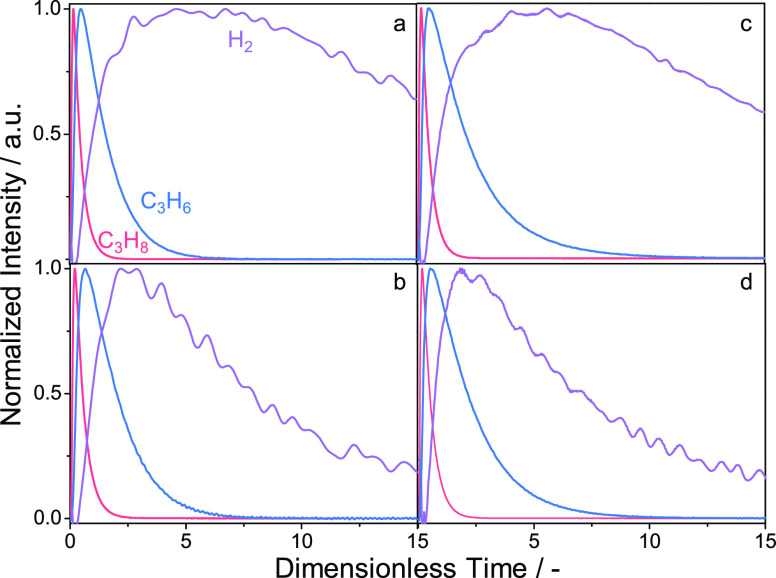
Height-normalized responses of C_3_H_8_, C_3_H_6_, and H_2_ after pulsing a C_3_H_8_:Ar = 1:1 mixture over (a) ZnO/ZrO_2_, (b) Zn_1_O_*x*_/TiZrO_*x*_, (c) Zn_1_O_*x*_-LaZrO_*x*_, and (d) Zn_1_O_*x*_-TiZrO_*x*_ at 550 °C. (a, b) Reprinted with permission from ref (4). Copyright 2020 American Chemical Society. (c, d) Reprinted with permission from ref (45). Copyright 2024 Elsevier.

Temporal analysis of products (TAP) has also been used to analyze the RDS in PDH due to its submillisecond resolution.^43,44^ The order of appearance (the time of maximum concentration (*t*_max_)) of reaction products after pulsing a C_3_H_8_-containing mixture is analyzed to draw conclusions about the RDS. Such tests with various ZnO_*x*_-based catalysts have shown that the *t*_max_ of H_2_ is significantly longer than the *t*_max_ of C_3_H_6_ (Figure 7). This is an indication of the lower rate of H_2_ formation in comparison with the rate of C_3_H_6_ formation. On this basis, the former step was concluded to be the RDS. It is also worth mentioning that the H_2_ response of Zn_1_O_*x*_/TiZrO_*x*_^4^ (Figure 7b) and Zn_1_O_*x*_-TiZrO_*x*_^45^ (Figure 7d) is less broad and has a lower *t*_max_ in compassion with the H_2_ response of ZnO/ZrO_2_^4^ (Figure 7a) and Zn_1_O_*x*_-LaZrO_*x*_^45^ (Figure 7c). Thus, the presence of Ti appears to facilitate the formation of gas-phase H_2_.

### 5.2 Effects of Support and ZnO_*x*_ Speciation on Catalyst Activity

**Figure 8:**
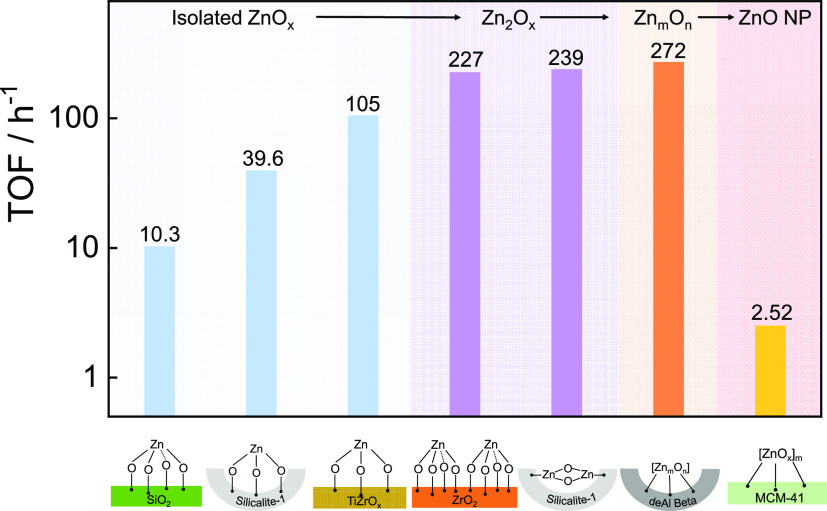
Intrinsic activity expressed as the Zn-related TOF of differently structured ZnO_*x*_ species in the PDH reaction at 550 °C using a feed with 40 vol % propane.

It is well known that the kind of support and metal/metal oxide speciation strongly affect the activity of catalysts in different reactions.^46^ To establish the structure–activity relationship in PDH over Zn-containing catalysts, we calculated the Zn-related turnover frequency (TOF) of propene formation under the assumption that each Zn atom is active.^1−4,28^ Since the catalysts were tested with different reaction feeds, we recalculated the reported TOF values under the assumption that the activity increases proportionally to the propane feed content (Figure 8). The reaction feed with 40vol% C_3_H_8_ was considered. The TOF value of Zn_1_O_*x*_ species on the surface of S-1 with a Zn–O CN of 3 is 39.6 h^–1^, which is about 4 times higher than that of Zn_1_O_*x*_ supported on amorphous SiO_2_ (Figure 8).^28^ Thus, the type of support should be an activity-determining factor. This assumption is further supported by a significantly higher TOF value (105 h^–1^) of isolated Zn_1_O_*x*_ on TiZrO_*x*_ (Figure 8).^4^ In addition, the nucleation degree of ZnO_*x*_ species should be noteworthy for the TOF. Zn_2_O_*x*_ species on the surface of S-1_1 or ZrO_2_ are similarly active (227 vs 239 h^–1^)^1^ but show about 6 times higher activity than Zn_1_O_*x*_ on the surface of S-1 (Figure 8).^3^ An even higher TOF of 272 h^–1^ was obtained over Zn_*m*_O_*n*_ clusters on the surface of deAl-Beta.^1^ Partially reduced ZnO NPs with an average Zn–Zn CN of 8.4 on MCM-41 have a TOF value of only 2.52 h^–1^.^3^

### 5.3 Effect of ZnO_*x*_ Speciation on Product Selectivity

**Figure 9:**
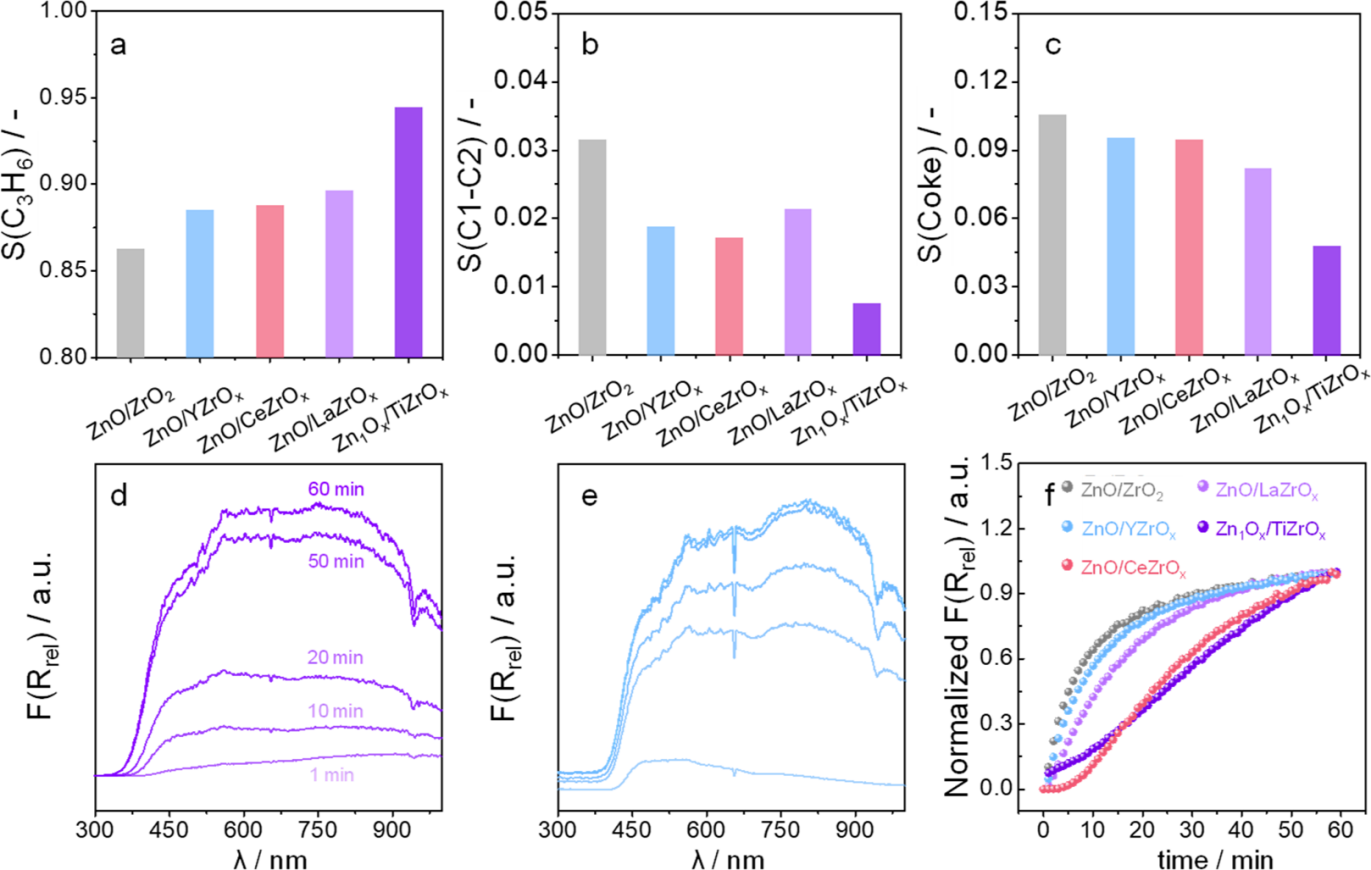
Dependence of selectivity to (a) propene, (b) cracking products, and (c) coke in PDH on the structure of ZnO_*x*_ species. Operando UV–vis spectra of reduced (d) Zn_1_O_*x*_/TiZrO_*x*_ and (e) ZnO/YZrO_*x*_. (f) Height-normalized F(R_rel_) at 800 nm after different times on a propane stream at 550 °C. WHSV(C_3_H_8_) was 3.21, 3.72, 4.71, 4.71, and 2.62 h^–1^ for ZnO/ZrO_2_, ZnO/LaZrO_*x*_, ZnO/YZrO_*x*_, Zn_1_O_*x*_/TiZrO_*x*_, and ZnO/CeZrO_*x*_, respectively. Reprinted with permission from ref (4). Copyright 2020 American Chemical Society.

Acidic sites on the surface of catalysts are generally accepted to be involved in the formation of carbon deposits in PDH and iBHD.^5^ Basic metal oxide promoters, e.g., K_2_O, are often introduced to neutralize the catalyst acidity and thus to increase propene selectivity.^5^ The structure of ZnO_*x*_ species can also influence coke formation as well as cracking reactions leading to CH_4_, C_2_H_4_, and C_2_H_6_ (C_1_–C_2_) as demonstrated in our study on Zn-containing catalysts based on ZrO_2_-containing supports.^4^ The space velocity was individually adjusted to achieve an initial propane conversion of 30% as required for a fair comparison of the catalysts. Zn_1_O_*x*_ species on TiZrO_*x*_ showed the highest propene selectivity of about 95% (Figure 9a) and the lowest selectivity to C_1_–C_2_ hydrocarbons (Figure 9b) and to coke (Figure 9c).

No correlation could be established between the selectivity to coke and the overall catalyst acidity determined by the temperature-programmed desorption of adsorbed NH_3_. However, the selectivity for these undesired products increased as the fraction of Zn_1_O_*x*_ decreased in favor of ZnO NPs. The effect of ZnO_*x*_ speciation on coke formation was elucidated by time-resolved operando UV–vis spectroscopic tests. Figure 9d,e exemplifies the UV–vis spectra expressed as the relative Kubelka–Munk function F(R_rel_) with respect to the spectrum of the coke-free catalyst. This function increases over the entire wavelength range due to the formation of carbon-containing deposits. The spectra could be roughly deconvoluted into three bands characteristic of low condensed (below 500 nm), medium condensed (500–800 nm), and highly condensed aromatics (above 800 nm). The temporal profiles of F(R_rel_) at 800 nm as a function of time of the propane stream are shown in Figure 9f. Their slope reflects the rate of coke formation. The larger the slope, the faster the rate. On this basis, the catalysts can be ordered as follows: ZnO/ZrO_2_ > ZnO/YZrO_*x*_ > ZnO/LaZrO_*x*_ > ZnO/CeZrO_*x*_ > Zn_1_O_*x*_/TiZrO_*x*_. Coke formation is initiated by the oligomerization of adsorbed propene molecules. Large ZnO NPs are favorable for achieving high coverage by such species due to the high density of neighboring sites involved in propene formation/adsorption.

### 5.4 Effect of Support Topology on Catalyst Performance

**Figure 10:**
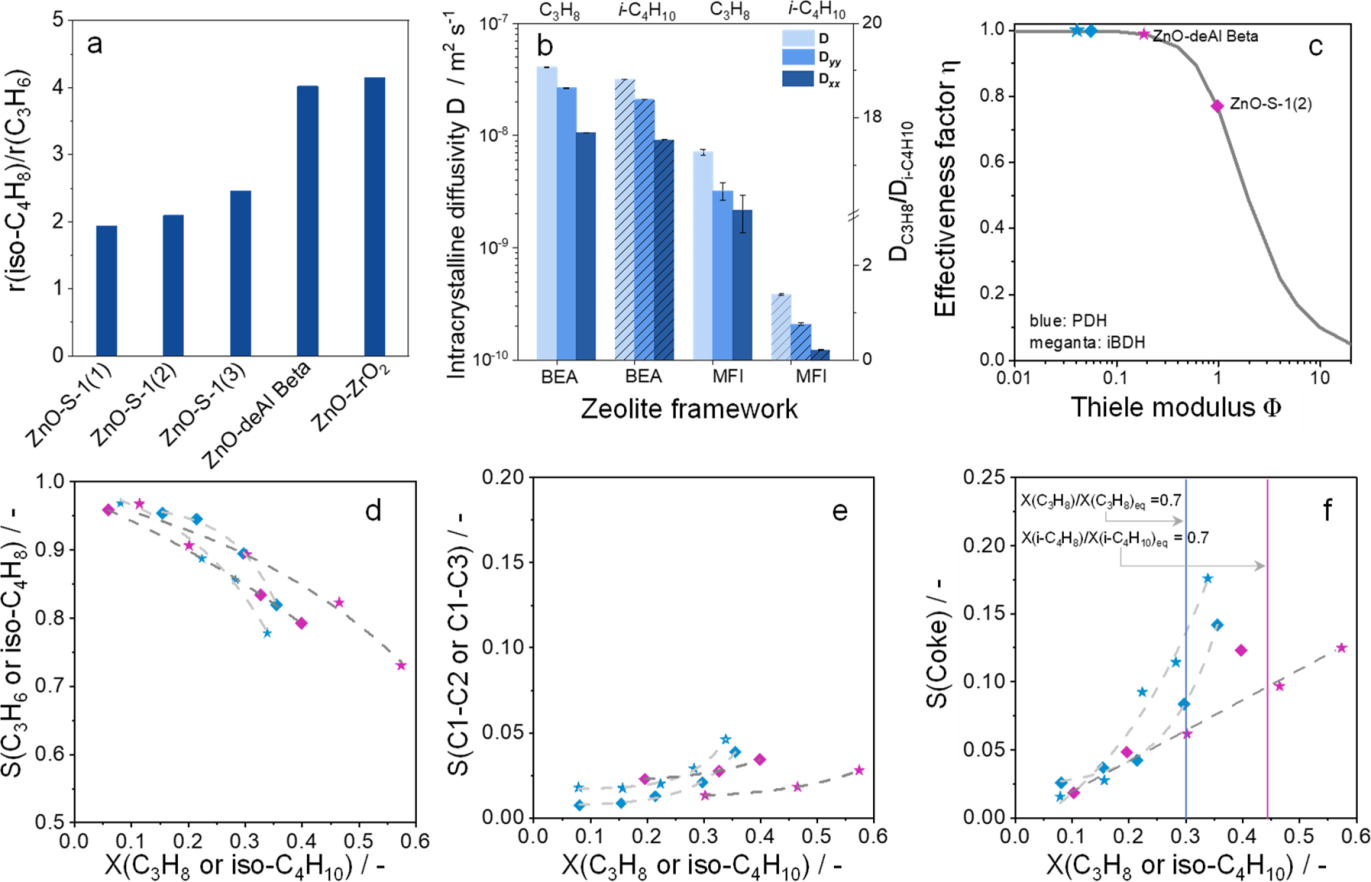
(a) Ratio of r(iso-C_4_H_8_) to r(C_3_H_8_). (b) Total diffusivity *D*, diffusivity along the straight channel *D*_*yy*_, and diffusivity along the curved channel *D*_*xx*_ of propane and isobutane within the BEA and MFI zeolite frameworks. (c) Effectiveness factor (η) of PDH and iBDH over different catalysts. Selectivity–conversion relationships for (d) propene (blue) and isobutene (magenta). (e) Cracking products (C_1_–C_2_ or C_1_–C_3_ hydrocarbons for PDH or iBDH). (f) Coke in PDH and iBDH over ZnO-S-1(2) and ZnO-deAl-Beta at 550 °C. In c–f, diamonds and stars represent ZnO-S-1(2) and ZnO-deAl-Beta, respectively. Reprinted with permission from ref (1). Copyright 2023 American Chemical Society.

Besides the ZnO_*x*_ speciation, support topology also affects catalyst activity and product distribution as concluded from PDH and iBDH tests using catalysts based on S-1, dealuminated Beta, and ZrO_2_ supports.^1^ The ratio of the rate of isobutene formation to that of propene formation (r(i-C_4_H_8_)/r(C_3_H_6_)) is about 2 for the ZnO-S-1 catalyst but about 4 for the ZnO-deAl-Beta and ZnO-ZrO_2_ catalysts, which have larger pores and an open structure, respectively (Figure 10a). These results suggest that the diffusion behavior of C_3_- and C_4_-hydrocarbons within the support pores should play an important role. This assumption was confirmed by the effectiveness factor (η = tanh ϕ/ϕ) calculated using the Thiele modulus ϕ which is defined as  because the DH reactions are first order with respect to alkane.^38,47,48^*D*, *l*, and *k* represent the intracrystalline diffusivity of alkane, the distance from the center to the surface of the zeolite along the straight channel, and the reaction rate constant, respectively. The latter was determined experimentally. The diffusivities of C_3_H_8_ and iso-C_4_H_10_ were calculated by force-field molecular dynamics (FFMD) simulations at 550 °C. The overall diffusivities of these hydrocarbons within deAl-Beta are very similar (Figure 10b). However, isobutane diffuses significantly more slowly within the S-1 zeolite. This difference affects η values, which are above 0.98 and below 0.8 for iBDH over ZnO-deAl-Beta and ZnO-S-1(2), respectively (Figure 10c).

To understand the effect of diffusion constraints on product distribution, selectivity–conversion relationships were obtained by varying the contact time (Figure 10d–f). Propene and isobutene are the primary products as their selectivity values are above zero at zero alkane conversion. The decrease in this selectivity with rising conversion suggests that these olefins are involved in consecutive reactions. Since the selectivity to cracking products and coke follows an opposite dependence, these products must be formed from the olefins. ZnO-S-1(2) showed higher propene selectivity and lower coke selectivity in PDH than did ZnO-deAl-Beta due to the presence of traces of acidic Al^3+^ cations in the latter support. These sites are responsible for coke deposits. However, ZnO-deAl-Beta revealed a higher isobutene selectivity and lower C_1_–C_3_ selectivity than did ZnO-S-1(2) in iBDH. The change in selectivity order was explained by diffusion constraints affecting secondary olefin transformations inside the zeolite pores. The diffusion of isobutene inside the deAl-Beta framework is more than 30 times faster than inside the S-1 framework, while there are no significant differences for the diffusion of propene.^1^ These results demonstrate that in addition to the known effect of mass-transport limitations on overall catalyst activity, they also affect the selectivity of cracking products in alkane DH reactions and thus contribute to a loss of selectivity to the desired olefins.

## 6 Conclusions and Perspectives

Heterogeneous catalysts with supported ZnO_*x*_ species have become state-of-the-art catalysts with industrially relevant performance in PDH and iBDH. The molecular structure of such a species is a key parameter affecting the catalyst performance. In this Account, we describe our recent achievements in the precise preparation of catalysts with uniform ZnO_*x*_ structures ranging from isolated sites, binuclear species, and subnanometer clusters to nanoparticles. On this basis, structure–activity–selectivity relationships have been established. This knowledge provides opportunities for the precise design and preparation of catalysts used not only for alkane dehydrogenation but also for alkane aromatization and CO_2_ hydrogenation to methanol, where ZnO_*x*_ is an active species or a promoter. We also identified the following areas of research in the development of DH catalysts that can find industrial applications.

(i) Many efforts have been devoted to the preparation of isolated ZnO_*x*_ species on various supports, while less information is available on the preparation of well-defined subnanometer Zn_*m*_O_*x*_ clusters. Such a gap should be closed as the clusters show significantly higher intrinsic activity than the isolated species. The development of methods to control the number of Zn atoms in the clusters is highly desirable to identify the optimal ZnO_*x*_ speciation.
(ii) Although the currently developed alkane dehydrogenation Zn-containing catalysts show promising propane productivity, the intrinsic activity of ZnO_*x*_ species should be further improved to reach the level of industrially used Pt-based catalysts. We expect that in addition to controlling ZnO_*x*_ speciation such improvements can be achieved through the use of promoters that accelerate RDS, as demonstrated by the example of ZnO_*x*_-TiZrO_*x*_ systems.^4^
(iii) The stability of ZnO_*x*_ species against reduction to metallic Zn at high reaction temperatures should be improved to avoid the loss of the metal and thus to improve catalyst on-stream stability. This can be achieved by strengthening the ZnO–support interactions or by introducing promoters. In addition, reaction engineering solutions for the capture/removal/storage of Zn^0^ and/or addition of fresh ZnO to the working catalyst should be considered.
(iv) In situ/operando characterization methods using cells resembling catalytic reactors are urgently needed to understand the structural changes of ZnO_*x*_ species under different conditions such as activation, reaction, deactivation, and regeneration. The methods should allow high spatial and temporal resolution. DFT calculations would complement the experiments to provide molecular-level details.

## Supporting Information Available

Data collected from the literature, calculated equilibrium conversion, and references (PDF)
