# Supplementary material for: Well-Defined Supported ZnOx Species: Synthesis, Structure, and Catalytic Performance in Nonoxidative Dehydrogenation of C3–C4 Alkanes
Source: Acc Chem Res. 2024 Apr 9;57(9):1264–74. doi: 10.1021/acs.accounts.4c00011 (PMC11080056; doi:10.1021/acs.accounts.4c00011)
Supplement: Supplementary file 1 — ar4c00011_si_002.pdf [file ar4c00011_si_002.pdf]

Supporting Information for

Well-defined Supported ZnO_x Species: Synthesis, Structure and Catalytic performance in Non-Oxidative Dehydrogenation of C_3-C_4 Alkanes

Shanlei Han[a], Dan Zhao[a], Evgenii V. Kondratenko[a]**

[a]*Leibniz-Institut für Katalyse e.V., Albert-Einstein-Str. 29a, 18059 Rostock, Germany*

**Correspondence to: dan.zhao@catalysis.de; evgenii.kondratenko@catalysis.de (E.V.K.)*

Table S1 Catalytic data of various materials tested in the non-oxidative propane dehydrogenation to propene under different reaction conditions.

Catalyst	Loading	T °C	WHSV h^{-1}	Feed	$X(C_3H_8)$ %	$S(C_3H_6)$ %	$X(C_3H_8)/X(C_3H_8)_{eq}$	STY $kg_{C3H6}\cdot kg_{Cat}^{-1}\cdot h^{-1}$	Reference
PtIn/Mg(Al)O-4	0.6 wt% Pt	620	3.3	C_3H_8:H_2:Ar=8:7:35	45.5	96.0	0.67	1.91	**1**
Pt/Sn2.00-Beta	0.538 wt% Pt	570	141.00	C_3H_8:H_2:N_2=10:10.80	50.0	98.0	0.94	65.95	2
PtSn/Al_2O_3 sheet	0.5 wt% Pt	590	9.40	C_3H_8:H_2:N_2=1:1.25:4	48.7	98.0	0.98	4.28	3
PtZn@S-1-Fin	0.45 wt% Pt	600	12.00	Pure C_3H_8	48.3	97.1	0.99	5.37	4
PtGa-Pb/SiO_2	3 wt% Pt	600	30.64	C_3H_8:H_2:He = 3.9:5:40	30.0	99.6	0.44	8.74	5
0.04Pt-0.36Zn-DeAlBEA	0.73 wt% Pt	550	215.66	C_3H_8:He = 25:75	18.2	99.0	0.37	37.05	6
PtMn/SiO_2	2.97 wt% Pt	550	33.86	C_3H_8:Ar = 10:40	40.7	92.5	0.77	12.17	7
0.7Pt0.7Zn/M-MFI	0.7 wt% Pt	580	9.10	Pure C_3H_8	35.0	98.0	0.85	2.98	8
PtZn@S-1	0.41 wt% Pt	600	5.90	C_3H_8:H_2:N_2 = 1:1:2	46.6	98.5	0.96	2.58	9
1Pt/1Ga-SBA-15	0.93 wt% Pt	580	20.00	C_3H_8:N_2 = 37:63	38.0	98.5	0.69	7.15	10
PtZnALD/SiO_2	1.15 wt% Pt	600	2.36	C_3H_8:He = 1:4	53.0	97.0	0.74	1.16	11
Pt-Sn-K/Al_2O_3	0.7 wt% Pt	620	4.00	C_3H_8:H_2 = 1:2	34.4	90.3	0.86	1.18	12
0.53Pt/0.68Sn-HMS	0.53 wt% Pt	550	37.71	C_3H_8:N_2 = 2:3	37.2	93.5	0.88	12.52	13
Pt/0.4Fe-DMSN	0.5 wt% Pt	590	2.40	C_3H_8:N_2 = 1:2	57.6	90.8	0.95	1.20	14
Seq-Imp Pt0.6Sn2.4/Al_2O_3	0.6 wt% Pt	550	70.71	C_3H_8:H_2:N_2 = 3:1:6	29.7	97.7	0.82	9.69	15
15% Zn−0.1% Pt/Al_2O_3	15 wt% Zn	600	3.00	C_3H_8:H_2 = 1:1	35.0	94.0	0.99	0.94	16
ZnO@NC/S-1(1.0)	2.0 wt% Zn	600	0.88	C_3H_8:H_2:N_2 = 1:1:5	56.4	85.0	0.94	0.40	17
Znβ-10	10 wt% Zn	600	0.40	C_3H_8:N_2 = 1:19	53.3	92.9	0.60	0.19	18
4Zn/TiZrOx	3.75 wt% Zn	550	4.71	C_3H_8:H_2:N_2 = 8:1:11	29.0	95.0	0.77	1.24	19
2Zn5.6ZrTi	2 wt% Zn	550	1.57	C_3H_8:H_2:N_2 = 2:1:2	23.2	95.0	0.84	0.33	20
Zn-4@S-1	4 wt% Zn	580	4.80	C_3H_8:N_2 = 1:9	30.0	96.0	0.40	1.32	21
$Zn_{0.15}Al_{2.56}O_4$	7.04 wt% Zn	600	2.48	C_3H_8:N_2 = 1:9	16.7	75.0	0.21	0.30	22

Catalyst	Loading	T °C	WHSV h^{-1}	Feed	$X(C_3H_8)$ %	$S(C_3H_6)$ %	$X(C_3H_8)/X(C_3H_8)eq$	STY $kg_{C3H6}\cdot kg_{Cat}^{-1}\cdot h^{-1}$	Reference
ZnO-S-1_3	8 wt% ZnO	550	7.90	$C_3H_8:N_2$ = 2:3	31.0	87.0	0.73	2.04	[23]
ZnO-S-1_3	8 wt% ZnO	600	15.70	$C_3H_8:N_2$ = 2:3	40.0	88.0	0.65	5.03	[23]
6ZnO/S-1	5.6 wt% Zn	550	2.36	$C_3H_8:N_2$ = 2:3	29.5	90.1	0.70	0.59	[24]
ZnO−deAl beta	8 wt% ZnO	550	7.69	$C_3H_8:N_2$ = 2:3	30.0	86.0	0.71	1.89	[25]
ZnO//$TiZrO_x$	2.40 wt% Zn	550	9.43	$C_3H_8:N_2$ = 2:3	0.2	0.96	0.51	1.99	[26]
ZnO//TiO_2	2.13 wt% Zn	550	2.83	$C_3H_8:N_2$ = 2:3	0.2	0.93	0.48	0.58	[26]
ZnO//$SiZrO_2$	3.11 wt% Zn	550	5.66	$C_3H_8:N_2$ = 2:3	0.2	0.92	0.52	1.21	[26]
ZnO//$LaZrO_x$	0.92 wt% Zn	550	5.66	$C_3H_8:N_2$ = 2:3	0.2	0.89	0.49	1.11	[26]
12V/γ-Al_2O_3	12 wt% V	600	3.00	$C_3H_8:H_2:N_2$ = 28:28:44	32.0	94.0	0.69	0.86	[27]
VO_x/γ-Al_2O_3	12 wt% V	600	8.25	$C_3H_8:H_2:N_2$ = 28:28:44	25.0	72.0	0.54	1.42	[28]
9V-MSNs	9 wt% V	600	1.18	C_3H_8:Ar = 1:9	50.6	87.5	0.63	0.50	[29]
8V-HMS	7.7 wt% V	550	3.14	$C_3H_8:N_2$ = 2:3	16.1	92.0	0.3	0.44	[30]
17.5Cr-2Ce-2K/Al	17.3 wt% Cr	630	11.79	$C_3H_8:N_2$ = 1:1	57.5	77.5	0.83	5.01	[31]
Cr/Al_2O_3-700	-	580	0.59	$C_3H_8:N_2$ = 1:19	25.0	97.0	0.30	0.14	[32]
Cr-Al-800	18 wt% Cr_2O_3	600	9.43	pure C_3H_8	33.2	90.4	0.68	2.70	[33]
2.5Cr-Ni/Al	2.5 wt% Cr	550	1.18	C_3H_8:Ar=1:9	47.0	95.0	0.74	0.50	[34]
Cr/SiO2-Al2O3(Si/Al=0.01)	20 wt% Cr_2O_3	630	2.85	$C_3H_8:N_2$=2:3	68.0	86.5	0.94	1.60	[35]
7.5Cr/Al2O3	7.5 wt% Cr	600	2.36	C_3H_8:Ar=1:4	62.2	89.0	0.87	1.25	[36]
5Si–5Co–Al_2O_3	5 wt% Co	590	3.18	$C_3H_8:H_2:N_2$ = 1:0.8:3.2	25.3	90.3	0.49	0.69	[37]
Co@S-1-steam	1 wt% Co	590	3.70	$C_3H_8:H_2:N_2$ = 5:4:5	20.7	96.0	0.52	0.70	[38]
Co/S-1	3 wt% Co	550	4.70	$C_3H_8:N_2$ = 1:4	34.4	95.3	0.65	1.47	[39]
Co@MFI-P50	3.14 wt% Co	600	7.50	pure C_3H_8	25.0	93.0	0.51	1.66	[40]
Co@S-1(EDA)	0.98 wt% Co	600	7.40	C_3H_8: Ar = 10: 33	37.1	93.0	0.54	2.44	[41]

Catalyst	Loading	T °C	WHSV h^{-1}	Feed	$X(C_3H_8)$ %	$S(C_3H_6)$ %	$X(C_3H_8)/X(C_3H_8)eq$	STY $kg_{C3H6}\cdot kg_{Cat}^{-1}\cdot h^{-1}$	Reference
Pt3GaK/Al_2O_3	3 wt% Ga	620	7.07	Pure C_3H_8	41.9	96.9	0.74	2.74	[42]
Pt/Ga/Al_2O_3	0.1 wt% Pt, 5 wt% Ga	580	2.36	C_3H_8:N_2=1:9	65.5	95.5	0.88	1.41	[43]
Ga-CHA(12, 1.0)	Ga/Al=1	550	5.89	C_3H_8:H_2:N_2 = 5:10:85	18.9	90.0	0.40	0.96	[44]
PC-PtGa	0.1 wt% Pt, 3.0 wt% Ga,	620	5.40	C_3H_8:He=2:8	58.5	98.0	0.75	2.96	[45]
2Ce-Pt/GaAL	0.1 wt% Pt, 3 wt% Ga	620	5.40	C_3H_8:He=2:8	60.0	96.0	0.77	2.97	[46]
5Ga-0.7Mg/ZSM-5	5.0 wt% Ga, 0.7 wt% Mg	600	4.70	C_3H_8:He=2:8	17.8	90.8	0.25	0.73	[47]

References:

(1) Xia, K.; Lang, W.-Z.; Li, P.-P.; Long, L.-L.; Yan, X.; Guo, Y.-J. The influences of Mg/Al molar ratio on the properties of PtIn/$MgAlO_x$ catalysts for propane dehydrogenation reaction. *Chem. Eng. J.* **2016**, *284*, 1068-1079.

(2) Xu, Z.; Yue, Y.; Bao, X.; Xie, Z.; Zhu, H. Propane Dehydrogenation over Pt Clusters Localized at the Sn Single-Site in Zeolite Framework. *ACS Catalysis* **2020**, *10* (1), 818-828.

(3) Shi, L.; Deng, G. M.; Li, W. C.; Miao, S.; Wang, Q. N.; Zhang, W. P.; Lu, A. H. Al_2O_3 Nanosheets Rich in Pentacoordinate Al^{3+} Ions Stabilize Pt-Sn Clusters for Propane Dehydrogenation. *Angew Chem Int Ed Engl* **2015**, *54* (47), 13994-13998.

(4) Zhang, B.; Li, G.; Liu, S.; Qin, Y.; Song, L.; Wang, L.; Zhang, X.; Liu, G. Boosting Propane Dehydrogenation over PtZn Encapsulated in an Epitaxial High-Crystallized Zeolite with a Low Surface Barrier. *ACS Catalysis* **2022**, *12* (2), 1310-1314.

(5) Nakaya, Y.; Hirayama, J.; Yamazoe, S.; Shimizu, K. I.; Furukawa, S. Single-atom Pt in intermetallics as an ultrastable and selective catalyst for propane dehydrogenation. *Nat Commun* **2020**, *11* (1), 2838.

(6) Qi, L.; Babucci, M.; Zhang, Y.; Lund, A.; Liu, L.; Li, J.; Chen, Y.; Hoffman, A. S.; Bare, S. R.; Han, Y.; et al. Propane Dehydrogenation Catalyzed by Isolated Pt Atoms in ≡SiOZn–OH Nests in Dealuminated Zeolite Beta. *J Am Chem Soc* **2021**, *143* (50), 21364-21378.

(7) Rochlitz, L.; Pessemesse, Q.; Fischer, J. W. A.; Klose, D.; Clark, A. H.; Plodinec, M.; Jeschke, G.; Payard, P. A.; Coperet, C. A Robust and Efficient Propane Dehydrogenation Catalyst from Unexpectedly Segregated Pt_2Mn Nanoparticles. *J Am Chem Soc* **2022**, *144* (29), 13384-13393.

(8) Han, S. W.; Park, H.; Han, J.; Kim, J.-C.; Lee, J.; Jo, C.; Ryoo, R. PtZn Intermetallic Compound Nanoparticles in Mesoporous Zeolite Exhibiting High Catalyst Durability for Propane Dehydrogenation. *ACS Catalysis* **2021**, *11* (15), 9233-9241.

(9) Zhang, B.; Li, G.; Zhai, Z.; Chen, D.; Tian, Y.; Yang, R.; Wang, L.; Zhang, X.; Liu, G. PtZn intermetallic nanoalloy encapsulated in silicalite-1 for propane dehydrogenation. *AIChE Journal* **2021**, *67* (7), e17295.

(10) Xin Li; Peng Rui; Wenfei Huang; Xin Yao; Yuewen Ye; Tongqi Ye; Morgan, D. J.; Carter, J. H. Propane Dehydrogenation Using Platinum Supported on Gallium-Doped Silica. *Catalysis Letters* **2024**, *154*, 634–642.

(11) Ingale, P.; Knemeyer, K.; Preikschas, P.; Ye, M.; Geske, M.; Naumann d'Alnoncourt, R.; Thomas, A.; Rosowski, F. Design of PtZn nanoalloy catalysts for propane dehydrogenation through interface tailoring via atomic layer deposition. *Catalysis Science & Technology* **2021**, *11* (2), 484-493.

(12) Zhang, H.; Wan, H.; Zhao, Y.; Wang, W. Effect of chlorine elimination from Pt-Sn catalyst on the behavior of hydrocarbon reconstruction in propane dehydrogenation. *Catalysis Today* **2019**, *330*, 85-91.

(13) Yuming Li; Yingjie Ma; Qiyang Zhang; Vita A. Kondratenko; Guiling Jiang; Huaqian Sun; Shanlei Han; Yajun Wang; Guoqing Cui; Mingxia Zhou; et al. Molecularly defined approach for preparation of ultrasmall Pt-Sn species for efficient dehydrogenation of propane to propene. *Journal of Catalysis* **2023**, *418*, 290-299.

(14) Liu, D.; Hu, H.; Yang, Y.; Cui, J.; Fan, X.; Zhao, Z.; Kong, L.; Xiao, X.; Xie, Z.

Restructuring effects of Pt and Fe in Pt/Fe-DMSN catalysts and their enhancement of propane dehydrogenation. *Catalysis Today* **2022**, *402*, 161-171.
(15) Gao, X.; Xu, W.; Li, X.; Cen, J.; Xu, Y.; Lin, L.; Yao, S. Non-oxidative dehydrogenation of propane to propene over Pt-Sn/Al_2O_3 catalysts: Identification of the nature of active site. *Chemical Engineering Journal* **2022**, *443*.
(16) Liu, G.; Zeng, L.; Zhao, Z.-J.; Tian, H.; Wu, T.; Gong, J. Platinum-Modified ZnO/Al_2O_3 for Propane Dehydrogenation: Minimized Platinum Usage and Improved Catalytic Stability. *ACS Catalysis* **2016**, *6* (4), 2158-2162.
(17) Zhao, D.; Li, Y.; Han, S.; Zhang, Y.; Jiang, G.; Wang, Y.; Guo, K.; Zhao, Z.; Xu, C.; Li, R.; et al. ZnO Nanoparticles Encapsulated in Nitrogen-Doped Carbon Material and Silicalite-1 Composites for Efficient Propane Dehydrogenation. *iScience* **2019**, *13*, 269-276.
(18) Chen, C.; Hu, Z.; Ren, J.; Zhang, S.; Wang, Z.; Yuan, Z. Y. ZnO Nanoclusters Supported on Dealuminated Zeolite β as a Novel Catalyst for Direct Dehydrogenation of Propane to Propylene. *ChemCatChem* **2019**, *11* (2), 868-877.
(19) Han, S.; Zhao, D.; Otroshchenko, T.; Lund, H.; Bentrup, U.; Kondratenko, V. A.; Rockstroh, N.; Bartling, S.; Doronkin, D. E.; Grunwaldt, J.-D.; et al. Elucidating the Nature of Active Sites and Fundamentals for their Creation in Zn-Containing ZrO_2–Based Catalysts for Nonoxidative Propane Dehydrogenation. *ACS Catalysis* **2020**, *10* (15), 8933-8949.
(20) Han, S.; Zhao, D.; Lund, H.; Rockstroh, N.; Bartling, S.; Doronkin, D. E.; Grunwaldt, J.-D.; Gao, M.; Jiang, G.; Kondratenko, E. V. TiO2-Supported catalysts with ZnO and ZrO_2 for non-oxidative dehydrogenation of propane: mechanistic analysis and application potential. *Catalysis Science & Technology* **2020**, *10* (20), 7046-7055.
(21) Xie, L.; Wang, R.; Chai, Y.; Weng, X.; Guan, N.; Li, L. Propane dehydrogenation catalyzed by in-situ partially reduced zinc cations confined in zeolites. *Journal of Energy Chemistry* **2021**, *63*, 262-269.
(22) Nadjafi, M.; Kierzkowska, A. M.; Armutlulu, A.; Verel, R.; Fedorov, A.; Abdala, P. M.; Müller, C. R. Correlating the Structural Evolution of ZnO/Al_2O_3 to Spinel Zinc Aluminate with its Catalytic Performance in Propane Dehydrogenation. *The Journal of Physical Chemistry C* **2021**, *125* (25), 14065-14074.
(23) Zhao, D.; Tian, X.; Doronkin, D. E.; Han, S.; Kondratenko, V. A.; Grunwaldt, J. D.; Perechodjuk, A.; Vuong, T. H.; Rabeah, J.; Eckelt, R.; et al. In situ formation of ZnO_x species for efficient propane dehydrogenation. *Nature* **2021**, *599* (7884), 234-238.
(24) Zhao, D.; Guo, K.; Han, S.; Doronkin, D. E.; Lund, H.; Li, J.; Grunwaldt, J.-D.; Zhao, Z.; Xu, C.; Jiang, G.; et al. Controlling Reaction-Induced Loss of Active Sites in ZnO_x/Silicalite-1 for Durable Nonoxidative Propane Dehydrogenation. *ACS Catalysis* **2022**, *12* (8), 4608-4617.
(25) Zhao, D.; Gao, M.; Tian, X.; Doronkin, D. E.; Han, S.; Grunwaldt, J.-D.; Rodemerck, U.; Linke, D.; Ye, M.; Jiang, G.; et al. Effect of Diffusion Constraints and ZnO_x Speciation on Nonoxidative Dehydrogenation of Propane and Isobutane over ZnO-Containing Catalysts. *ACS Catalysis* **2023**, *13* (5), 3356-3369.
(26) Dan Zhao; Vita A. Kondratenko; Dmitry E. Doronkin; Shanlei Han; Jan-Dierk Grunwaldt; Uwe Rodemerck; David Linke; Kondratenko, E. V. Effect of supports on the kind of in-situ formed ZnO_x species and its consequence for non-oxidative propane dehydrogenation. *Catalysis Today* **2024**, *402*, 310-318.
(27) Liu, G.; Zhao, Z.-J.; Wu, T.; Zeng, L.; Gong, J. Nature of the Active Sites of VO_x/Al_2O_3

Catalysts for Propane Dehydrogenation. *ACS Catalysis* **2016**, *6* (8), 5207-5214.
(28) Zhao, Z. J.; Wu, T.; Xiong, C.; Sun, G.; Mu, R.; Zeng, L.; Gong, J. Hydroxyl-Mediated Non-oxidative Propane Dehydrogenation over VO_x/gamma-Al_2O_3 Catalysts with Improved Stability. *Angew Chem Int Ed Engl* **2018**, *57* (23), 6791-6795.
(29) Wu, Y.-L.; Han, Z.-F.; Yan, X.; Lang, W.-Z.; Guo, Y.-J. Effective synthesis of vanadium-doped mesoporous silica nanospheres by sol-gel method for propane dehydrogenation reaction. *Microporous and Mesoporous Materials* **2022**, *330*, 111616.
(30) Li, Y.; Yu, X.; Zhang, Q.; Kondratenko, V. A.; Wang, Y.; Cui, G.; Zhou, M.; Xu, C.; Kondratenko, E. V.; Jiang, G. The nature of VOx structures in HMS supported vanadium catalysts for non-oxidative propane dehydrogenation. *Journal of Catalysis* **2022**, *413*, 658-667.
(31) Kang, K. H.; Kim, T. H.; Choi, W. C.; Park, Y.-K.; Hong, U. G.; Park, D. S.; Kim, C.-J.; Song, I. K. Dehydrogenation of propane to propylene over CrO_y-CeO_2-K_2O/γ-Al_2O_3 catalysts: Effect of cerium content. *Catalysis Communications* **2015**, *72*, 68-72.
(32) Hu, Z.-P.; Wang, Z.; Yuan, Z.-Y. Cr/Al_2O_3 catalysts with strong metal-support interactions for stable catalytic dehydrogenation of propane to propylene. *Molecular Catalysis* **2020**, *493,* 111052.
(33) Xin-Qian Gao; Wen-Duo Lu; Shou-Zhao Hu; Wen-Cui Li; Lu, A.-H. Rod-shaped porous alumina-supported Cr_2O_3 catalyst with low acidity for propane dehydrogenation. *Chinese Journal of Catalysis* **2019**, *40* (2), 184-191.
(34) Li, P.-P.; Lang, W.-Z.; Xia, K.; Luan, L.; Yan, X.; Guo, Y.-J. The promotion effects of Ni on the properties of Cr/Al catalysts for propane dehydrogenation reaction. *Applied Catalysis A: General* **2016**, *522*, 172-179.
(35) Hamid Karami; Saeed Soltanali; Amir Mohammad Najafi; Maryam Ghazimoradi; Elham Yaghoobpour; Abbasi, A. Amorphous silica-alumina as robust support for catalytic dehydrogenation of propane: Effect of Si/Al ratio on nature and dispersion of Cr active sites. *Applied Catalysis A: General* **2023**, *658*, 119167.
(36) Lang, W.-Z.; Hu, C.-L.; Chu, L.-F.; Guo, Y.-J. Hydrothermally prepared chromia-alumina (xCr/Al_2O_3) catalysts with hierarchical structure for propane dehydrogenation. *RSC Adv.* **2014**, *4* (70), 37107-37113.
(37) Dai, Y.; Wu, Y.; Dai, H.; Gao, X.; Tian, S.; Gu, J.; Yi, X.; Zheng, A.; Yang, Y. Effect of coking and propylene adsorption on enhanced stability for Co^{2+}-catalyzed propane dehydrogenation. *Journal of Catalysis* **2021**, *395*, 105-116.
(38) Jiangping Long; Suyang Tian; Sheng Wei; Hongqiao Lin; Guiwen Shi; Xupeng Zong; Yanhui Yang; Dan Yang; Yu Tang; Dai, Y. Direct dehydrogenation of propane over Co@silicalite-1 zeolite: Steaming-induced restructuring of Co^{2+} active sites. *Applied Surface Science* **2023**, *614*, 156238.
(39) Yuming Li; Qiyang Zhang; Shuting Fu; Vita A. Kondratenko; Tatiana Otroshchenko; Stephan Bartling; Yaoyuan Zhang; Anna Zanina; Yajun Wang; Guoqing Cui; et al. Active species and fundamentals of their creation in Co-containing catalysts for efficient propane dehydrogenation to propylene. *Chemical Engineering Journal* **2023**, *460*, 141778.
(40) Lv, X.; Yang, M.; Song, S.; Xia, M.; Li, J.; Wei, Y.; Xu, C.; Song, W.; Liu, J. Boosting Propane Dehydrogenation by the Regioselective Distribution of Subnanometric CoO Clusters in MFI Zeolite Nanosheets. *ACS Appl Mater Interfaces* **2023**, 15(11), 14250–14260
(41) Liu, Y.; Lu, S.; Yuan, T.; Wang, S.; Bahnemann, J.; Jiang, F.; Chen, H. Uniformly stable

hydroxylated cobalt(II) silicate species embedded within silicalite-1 zeolite for boosting propane dehydrogenation. *Microporous and Mesoporous Materials* **2023**, *352*, 112516.
(42) Sattler, J. J.; Gonzalez-Jimenez, I. D.; Luo, L.; Stears, B. A.; Malek, A.; Barton, D. G.; Kilos, B. A.; Kaminsky, M. P.; Verhoeven, T. W.; Koers, E. J.; et al. Platinum-promoted Ga/Al_2O_3 as highly active, selective, and stable catalyst for the dehydrogenation of propane. *Angew Chem Int Ed Engl* **2014**, *53* (35), 9251-9256.
(43) Yu, Q.; Yu, T.; Chen, H.; Fang, G.; Pan, X.; Bao, X. The effect of Al^{3+} coordination structure on the propane dehydrogenation activity of Pt/Ga/Al_2O_3 catalysts. *Journal of Energy Chemistry* **2020**, *41*, 93-99.
(44) Yuan, Y.; Lee, J. S.; Lobo, R. F. Ga^+-Chabazite Zeolite: A Highly Selective Catalyst for Nonoxidative Propane Dehydrogenation. *J Am Chem Soc* **2022**, *144* (33), 15079-15092.
(45) Im, J.; Choi, M. Physicochemical Stabilization of Pt against Sintering for a Dehydrogenation Catalyst with High Activity, Selectivity, and Durability. *ACS Catalysis* **2016**, *6* (5), 2819-2826.
(46) Kwon, H. C.; Park, Y.; Park, J. Y.; Ryoo, R.; Shin, H.; Choi, M. Catalytic Interplay of Ga, Pt, and Ce on the Alumina Surface Enabling High Activity, Selectivity, and Stability in Propane Dehydrogenation. *ACS Catalysis* **2021**, *11* (17), 10767-10777.
(47) Yang, G.; Yan, X.; Chen, Y.; Guo, X.-J.; Lang, W.-Z.; Guo, Y.-J. Improved propylene selectivity and superior catalytic performance of Ga-xMg/ZSM-5 catalysts for propane dehydrogenation (PDH) reaction. *Applied Catalysis A: General* **2022**, *643*, 118778
